# MicroRNA 146a is associated with diabetic complications in type 1 diabetic patients from the EURODIAB PCS

**DOI:** 10.1186/s12967-021-03142-4

**Published:** 2021-11-25

**Authors:** Federica Barutta, Beatrice Corbetta, Stefania Bellini, Simonetta Guarrera, Giuseppe Matullo, Michela Scandella, Casper Schalkwijk, Coen D. Stehouwer, Nish Chaturvedi, Sabita S. Soedamah-Muthu, Marilena Durazzo, Gabriella Gruden

**Affiliations:** 1 Diabetic Nephropathy Laboratory, Department of Medical Sciences, University of Turin, C/so Dogliotti 14, 10126 Turin, Italy; 2 Italian Institute for Genomic Medicine, IIGM, Candiolo, Italy; 3 Candiolo Cancer Institute, FPO-IRCCS, Candiolo, Italy; 4 Medical Genetics Unit, AOU Città Della Salute E Della Scienza, Turin, Italy; 5 Department of Internal Medicine and Cardiovascular Research Institute Maastricht (CARIM), Maastricht University, Maastricht, The Netherlands; 6 Institute of Cardiovascular Science, University College London, London, UK; 7 Center of Research On Psychology in Somatic Diseases (CORPS), Department of Medical and Clinical Psychology, Tilburg University, Tilburg, The Netherlands; 8 Institute for Food, Nutrition and Health, University of Reading, Reading, UK

**Keywords:** MicroRNAs, Type 1 diabetes, Retinopathy, Cardiovascular diseases

## Abstract

**Background:**

MicroRNA-146a-5p (miR-146a-5p) is a key regulator of inflammatory processes. Expression of miR-146a-5p is altered in target organs of diabetic complications and deficiency of miR-146a-5p has been implicated in their pathogenesis. We investigated if serum miR-146a-5p levels were independently associated with micro/macrovascular complications of type 1 diabetes (DM1).

**Methods:**

A nested case–control study from the EURODIAB PCS of 447 DM1 patients was performed. Cases (n = 294) had one or more complications of diabetes, whereas controls (n = 153) did not have any complication. Total RNA was isolated from all subjects and miR-146a-5p levels measured by qPCR. Both the endogenous controls U6 snRNA and the spike (Cel-miR-39) were used to normalize the results. Logistic regression analysis was carried out to investigate the association of miR-146a-5p with diabetes complications.

**Results:**

MiR-146a-5p levels were significantly lower in cases [1.15 (0.32–3.34)] compared to controls [1.74 (0.44–6.74) P = 0.039]. Logistic regression analysis showed that levels of miR-146a-5p in the upper quartile were inversely associated with reduced odds ratio (OR) of all complications (OR 0.34 [95% CI 0.14–0.76]) and particularly with cardiovascular diseases (CVD) (OR 0.31 [95% CI 0.11–0.84]) and diabetic retinopathy (OR 0.40 [95% CI 0.16–0.99]), independently of age, sex, diabetes duration, A1c, hypertension, AER, eGFR, NT-proBNP, and TNF-α.

**Conclusions:**

In this large cohort of DM1 patients, we reported an inverse and independent association of miR-146a-5p with diabetes chronic complications and in particular with CVD and retinopathy, suggesting that miR-146a-5p may be a novel candidate biomarker of DM1 complications.

**Supplementary Information:**

The online version contains supplementary material available at 10.1186/s12967-021-03142-4.

## Background

Patients with type 1 diabetes mellitus (DM1) are at high risk of developing micro (retinopathy, nephropathy, neuropathy) and macrovascular complications of diabetes. Discovery of new biomarkers of complications is crucial to improve prediction, early diagnosis, and prognosis.

There is increasing evidence that abnormally expressed microRNAs (miRNAs) in target organs of diabetic complications are involved in the pathogenesis of vascular complications of diabetes [1–3]. MiRNAs are also present in body fluids and circulating miRNAs hold good promise as non-invasive clinical biomarkers. Indeed, serum miRNAs are very stable as they are either packaged in extracellular vesicles (EVs) or bound to proteins that protect them from endogenous RNases activity [4–7].

There is relatively poor knowledge on circulating miRNAs as biomarkers in the context of DM1 complications [2, 8–10]. However, serum levels of a panel of specific TGF-β1-regulated miRNAs predict progression to end-stage renal failure (ESRF) in DM1 patients with proteinuria [9]. Moreover, circulating miR-27b, miR-320a, and miR-126 levels were found independently associated with diabetic retinopathy [11, 12].

miR-146a-5p is a key negative regulator of inflammatory processes. Specifically, NF-kB, a pro-inflammatory transcription factor, induces miR-146a-5p expression, which in turn represses target genes important in NF-kB activation [13, 14]. Therefore, miR-146a-5p expression is a mechanism by which NF-κB restrains its inflammation-promoting activity and favors resolution of inflammation. In addition, miR-146a-5p prevents inflammasome activation and macrophage shift to the proinflammatory M1 phenotype [15]. Preclinical studies have shown that insufficient miR-146a-5p expression fuels inflammation thereby contributing to the pathogenesis of diabetes vascular complications [15–19].

Whether serum miR-146a-5p levels are associated with vascular complications of DM1 diabetes is unknown; however, miR-146a-5p was one of the 25 differentially expressed miRNAs in a profiling analysis performed in pooled serum samples from DM1 patients with and without complications [20].

The aim of the present study was to confirm these profiling results and to assess if elevated miR-146a-5p levels affected odds ratios (ORs) of micro/macrovascular complications in DM1 patients from the EURODIAB PCS nested case–control study.

## Methods

### Patient sample

The EURODIAB Type 1 Diabetes Complications Study (1989–1991) was designed to discover risk factors of diabetes complications in 3,250 subjects with DM1[21]. Seven years later study participants were invited for re-examination in the EURODIAB Prospective Complication Study (PCS 1997–1999) [22] and a nested case–control study was designed at follow-up. Cases had at least one complication among cardiovascular diseases (CVD), retinopathy, and albuminuria; whereas controls were free of complications [23–26]. The present study included 460 subjects (300 cases and 160 controls, ratio 1.9) with available serum samples and data on complications. Cases and controls were unmatched and adjustments were made at the analysis stage. Risk factors [cholesterol, hypertension, body mass index (BMI), triglycerides, A1c] were assessed as previously described [26]. Retinopathy was evaluated and graded from retinal photographs using the EURODIAB protocol [22]. Albumin excretion rate (AER) was measured on two 24-h urine collections by immunoturbidimetry and classified as normo- (< 20 μg/min), micro- (20–200 μg/min), or macro-albuminuria (≥ 200 μg/min). Glomerular filtration rate (eGFR) was estimated using the four-component equation from the Modification of Diet in Renal Disease study [27]. CVD was defined as a physician-diagnosed positive history of myocardial infarction, coronary artery bypass graft surgery, angina, stroke and/or ischemic changes on a centrally Minnesota-coded EKG.

The study was approved by the Ethical Committee of Turin University. The procedures were in accordance with the Helsinki Declaration. Written consent has been obtained from each patient or subject after full explanation of the purpose and nature of all procedures used.

### Biomarkers

Tumor necrosis factor α (TNF-α) and interleukin 6 (IL-6) levels were measured by ELISA assays (R&D Systems) as described previously [23, 26]. NT-proBNP was measured by two-site sandwich electrochemiluminescence immunoassay (Elecsys proBNP II; Roche, Mannheim, Germany) [25].

### RNA isolation

Total RNA was prepared using the Trizol®LS reagent (Thermo Fisher, Milan, Italy) according to the manufacturer’s recommendations. In brief, 200 µl of serum was mixed thoroughly with 750 µl of TRIZOL®LS reagent. Mixtures were then gently inverted 5–8 times, and incubated at room temperature (RT) for 15 min. After which, both the spike-in Cel-miR-39 (3 µl) and 200 µl of chloroform were added and the solution was mixed vigorously. The samples were then centrifuged at 12,000xg for 15 min at 4 °C, the upper aqueous phase was carefully transferred to a new tube, upon which 500 µl isopropanol was added, and then incubated for 10 min at RT, before then being centrifuged a 4 °C, 12,000xg for 10 min. Pellets were washed with 75% ethanol, air-dried at RT for 10 min, and re-suspended in 25 μl of nuclease free H_2_O. RNA quality was assessed by capillary electrophoresis on an Agilent-2100 Bioanalyzer (Agilent Technologies, Santa Clara, CA).

### Reverse transcription (RT) and pre-amplification

RT reaction was performed by using TaqMan MicroRNA Reverse Transcription Kit according to the company’s recommendations. Three µl of RNA solution from the 25 µl eluate was combined with 6 µl of RT primer pool, 0.30 µl of dNTPs with dTTP (100 mmol/L), 1.50 µl of 10 × RT Buffer, 3 µl of Multiscribe Reverse Transcriptase and 0.19 µl of RNase (20U/µl) to a final volume of 15 µl. The RT-PCR reaction was set as follows: 16 °C for 30 min, 42 °C for 30 min and 85 °C for 5 min using a Veriti thermocycler (Thermo Fisher, Milan, Italy). The RT reaction products were further amplified using the Megaplex PreAmp Primers (Thermo Fisher, Milan, Italy). A 2.5 µl aliquot of the RT reaction product was combined with 12.5 µl of Pre-amplification Mastermix (2x) and 3.75 µl of Preamplification primer pool (10x) to a final volume of 25 µl. The pre-amplification reaction was performed by heating the samples at 95 °C for 10 min, followed by 12 cycles of 95 °C for 15 s and 60 °C for 4 min. Finally, samples were heated at 99.9 °C for 10 min to ensure enzyme inactivation. Pre-amplification reaction products were diluted to a final volume of 100 µl and stored at − 20 °C.

### Taqman qPCR assay

qPCR was performed using Taqman reagents (Taqman miRNA Assay, Taqman Universal PCR Master Mix No AmpErase UNG). Diluted pre-amplification products (0.10 µl) were combined with 0.50 µl of Taqman miRNA Assay (20x) and 5 µl of the Taqman Universal PCR Master Mix No AmpErase UNG (2x) to a final volume of 10 µl. qPCR was performed on an Applied Biosystems 7900HT thermocycler at 95 °C for 10 min, followed by 40 cycles of 95 °C for 15 s and 60 °C for 1 min. The comparative Ct method (2^−ΔΔCt^). (SDS2.2 software) was used to calculate relative expression. Results were normalized using both the endogenous control U6 snRNA and the spike in Cel-miR-39. Thirteen samples with Ct values ≥ 35 or undetermined for Cel-miR-39, miR-146a-5p and U6 ≥ 35 were excluded from the analyses.

### Statistical analyses

Normally distributed variables are reported as means (standard deviation, SD), while non-normally distributed variables were log-transformed (miR-146a-5p, triglycerides, NT-proBNP, TNF-α, IL6, AER) and reported as geometric means (25°-75° percentiles). Student's t-test and ANOVA were used for comparisons. Categorical variables were compared using the Chi-Squared test. Pearson’s correlation coefficient analysis was used to assess the relationship between miR-146a-5p and clinical variables. Logistic regression analysis was employed to estimate the odds ratios (ORs) of miR-146a-5p for all chronic complications (micro-macroalbuminuria, CVD, retinopathy), independently of established risk factors and confounders. We used the likelihood ratio test to compare nested models, exploring the relevance of age, gender, diabetes duration, BMI, A1c, blood pressure, total cholesterol, AER, eGFR, NT-proBNP, IL-6, TNF-α, and smoking. Analyses were hypothesis-oriented and variables were retained in the final model if they added significantly to the likelihood of models or to the estimated coefficients of predictors. As miR-146a-5p may play a different role in various complications, models were also fitted for each complication separately. To assess ORs for increasing miR-146a-5p levels, miR-146a-5p levels were categorized by quartile distribution in controls. Because ORs in the three lower quartiles were similar, they were combined as the reference category in the final analysis and compared to the upper quartile. A p value < 0.05 was considered statistically significant. Analyses were performed using the SPSS (Version 27) software.

## Results

### Patients’ clinical characteristics

**Fig. 1:**
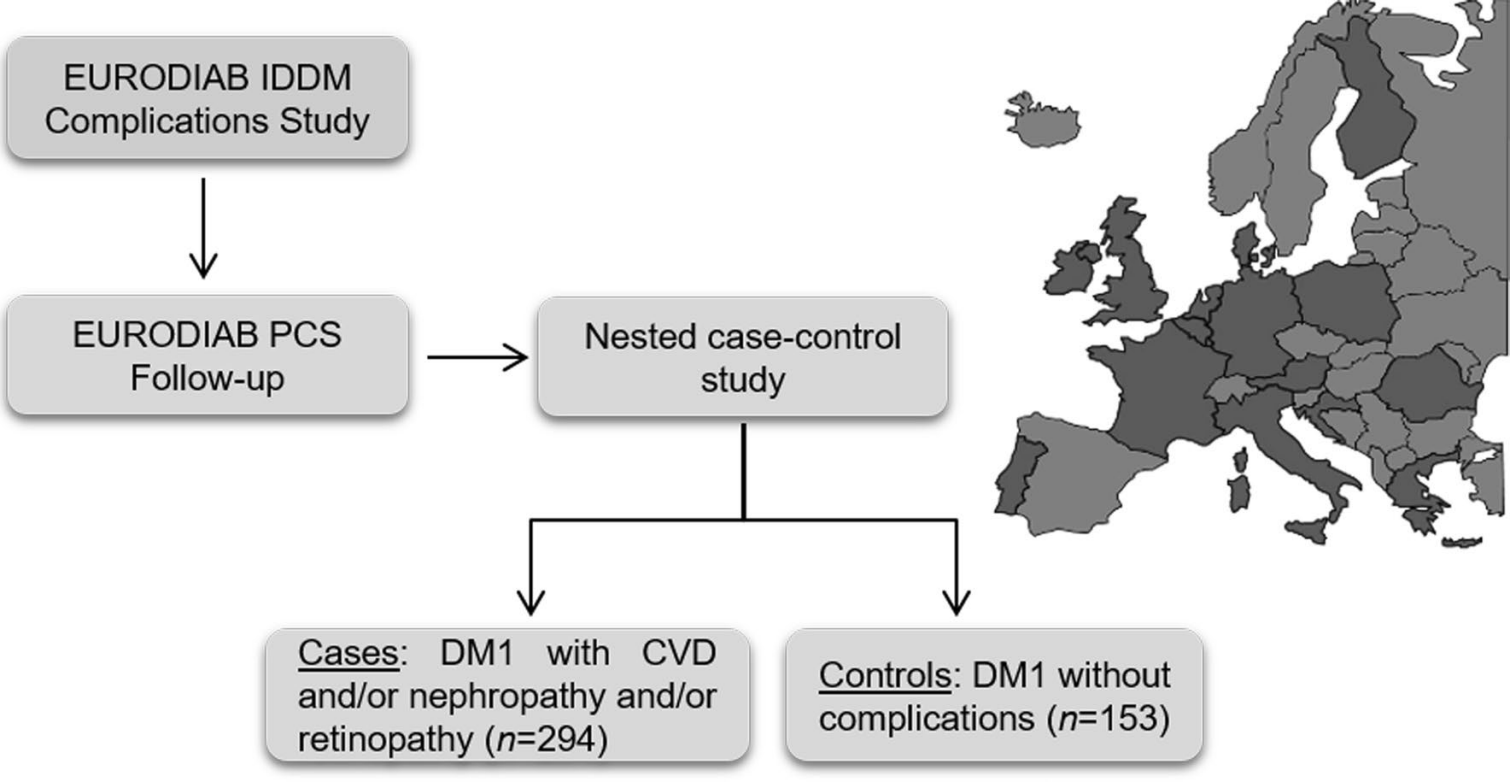
Flow chart for the study population. The map shows the European countries that participated to the EURODIAB study (dark grey)

The studied population (n = 447) (Fig. 1) had an average age of 39.5 ± 10.1 years, mean diabetes duration of 21.6 ± 9.7 years, and a similar percentage of men and women. Among the 294 cases, 179 individuals had nephropathy (40.8% micro- and 59.2% macroalbuminuria) and 257 individuals had retinopathy (background 48.6% and proliferative 51.4%). However, most subjects 165 (56.1%) had both microvascular complications. CVD was present in 117 subjects (39.8%) and most of them had CAD (90.6%). Table 1 shows the characteristics of both cases and controls. Overall cases had a worse risk factor profile than controls, as shown by more unfavorable levels of conventional risk factors and inflammatory cytokines.

**Table 1:** Characteristics of the individuals recruited in the Nested Case–Control Study of the EURODIAB PCS

	Case subjects *(n* = *294)*	Control subjects *(n* = *153)*	P value
Age (years)	41.60 ± 10.70	35.50 ± 7.50	< 0.001
Sex (M/F%)	53/47	48/52	0.35
BMI (Kg/m^2^)	24.80 ± 3.60	23.80 ± 2.60	0.001
Diabetes duration (years)	24.95 ± 9.23	15.31 ± 7.12	< 0.001
A1C (%)	9.02 ± 1.61	7.77 ± 1.19	< 0.001
Systolic BP (mmHg)	127.30 ± 22.20	114.60 ± 13.50	< 0.001
Diastolic BP (mmHg)	75.90 ± 11.40	73.50 ± 10.80	0.030
Current smoker (%)	32.90	28.10	0.01
Hypertension (%)	55.80	13.40	< 0.001
eGFR (ml/min/1.73m^2^)	90.31 ± 25.04	106.20 ± 13.74	< 0.001
Total cholesterol (mmol/l)	5.48 ± 1.23	4.90 ± 1.09	< 0.001
LDL-cholesterol (mmol/l)	3.35 ± 1.11	2.79 ± 0.97	< 0.001
HDL-cholesterol (mmol/l)	1.60 ± 0.42	1.66 ± 0.40	0.157
Triglycerides (mmol/l)	1.19 (0.82–1.58)	0.82 (0.64–1.08)	< 0.001
NT-proBNP (pg/ml)	76.31 (31.34–133.50)	41.10 (22.57–75.48)	< 0.001
TNF-α (pg/ml)	3.27 (2.40–4.30)	2.21 (1.72–2.85)	< 0.001
IL-6 (pg/ml)	2.50 (1.33–3.79)	1.80 (1.17–2.55)	< 0.001
miR-146a-5p	1.15 (0.32–3.34)	1.74 (0.44–6.73)	0.039

Data are expressed as mean ± SD, percentage or geometric mean (25°-75° percentile) for log-transformed data. BMI, body mass index; LDL, low-density lipoprotein; HDL, high-density lipoprotein; BP, blood pressure; eGFR, estimated glomerular filtration rate; NT-proBNP, NT-proB-type Natriuretic Peptide; IL-6, interleukin 6; TNF-α, tumor necrosis factor alpha

### miR-146a-5p expression

MiR-146a-5p was measurable in all the 447 samples and value distribution was right-skewed. Individual Ct values are reported in the Additional file 1: Table S1. There were no differences in mir146a-5p levels between men and women [men: 1.32 (0.37–4.53); women: 1.34 (0.33–4.20), p = 0.91] and across age categories [≤ 35.0: 1.35 (0.38–4.31); 35.1–45.9: 1.32 (0.32–4.23); 46.0–55.9: 1.26 (0.26–5.06) and ≥ 56.0 years: 1.34 (0.46–3.53), p = 0.95].

### Correlation of miR-146a-5p with clinical parameters and serum biomarkers

We then explored the association between miR-146a-5p and a range of relevant biochemical and clinical variables. Correlations of all variables in a matrix are reported in Additional file 2: Table S2. There was no correlation between miR-146a-5p and age even after adjustment for sex (p = 0.823). Similarly, no correlation was found between miR-146a-5p levels and diabetes duration neither in the whole population nor in cases (cases: r = 0.038, p = 0.521). Notably, serum miR-146a-5p levels were inversely correlated with diastolic blood pressure (*r* = -0.11, *p* = 0.019), triglycerides (*r* = -0.12, *p* = 0.01), TNF-α (*r* = -0.17, *p* = 0.000), and NT-proBNP (*r* = -0.15, *p* = 0.002). Moreover, miR-146a-5p levels were found to be positively correlated with renal function (eGFR *r* = 0.22, *p* = 0.000).

### MiR-146a-5p levels in cases vs. controls

**Fig. 2:**
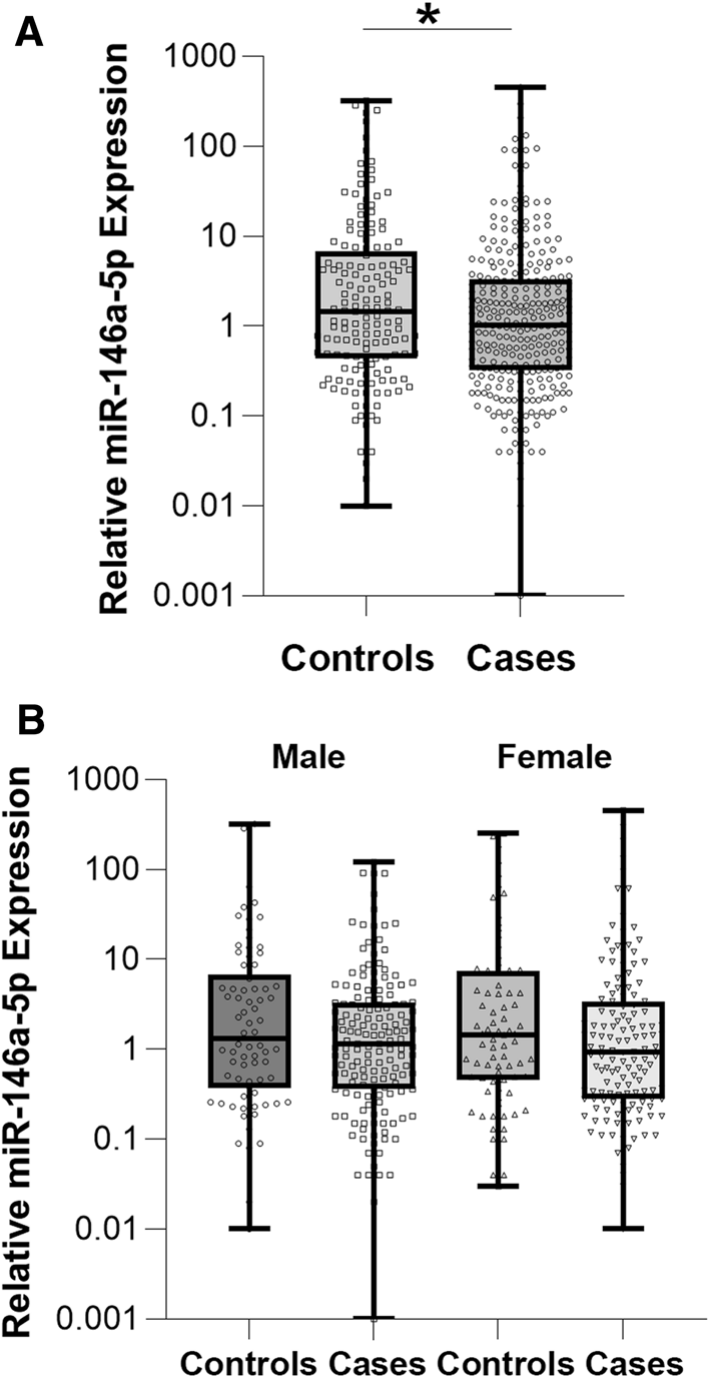
miR-146a-5p expression. **A** Comparison of miR-146a-5p levels in the serum of DM1 patients with (cases; n = 294) and without (controls; n = 153) micro-macrovascular complications (*p < 0.05 cases vs. controls). **B** Gender-specific miR-146a-5p expression in both cases and controls (p = ns)

Levels of miR-146a-5p were significantly lower in cases [1.15 (0.32–3.32)] compared to controls [1.74 (0.44–6.11) P = 0.039] (Fig. 2A), and results were not modified after adjustment for age and sex (P = 0.042) and diabetes duration (P = 0.049). Among cases miR-146a-5p levels were similar in patients with one [1.13 (0.31–3.00)] or multiple (> 1) [1.15 (0.33–3.38)] diabetes complications. When the comparison was performed separately in each sex, miR-146a-5p levels were still lower in cases compared to controls in both men and women; but differences did not reach statistical significance [men; cases: 1.16 (0.36–3.31); controls: 1.72 (0.36–6.77), P = 0.160; women: cases 1.15 (0.28–3.39); controls: 1.76 (0.46–7.34), P = 0.209] (Fig. 2B).

### Logistic regression

Logistic regression analyses were carried out to establish if miR-146a-5p changed the odds ratios (OR) of having either all or individual vascular complications, independently of risk factors and potential confounders. Levels of miR-146a-5p in the upper quartile (≥ 6.73) were associated with a 59% lower risk of having all complications (OR = 0.41, 95% CI 0.21–0.79) and this was independent of age, gender, A1c, and duration of diabetes (Model 1-Table 2). After adjustment for AER, hypertension, eGFR, smoking, the strength of this inverse association was even greater (OR = 0.32, 95% CI 0.14–0.73) (Model 2-Table 2). Following further adjustment for TNF-α and NT-proBNP, miR-146a-5p values in the upper quartile are still reduced by 66% the OR for all complications (OR = 0.34, 95% CI 0.14–0.76) (Model 3-Table 2). Models were not affected by the inclusion of total cholesterol, BMI and IL-6. Analysis performed separately in men and women showed that miR-146a-5p was inversely and independently associated with all complications in men. An inverse association was also observed in women, but did not reach statistical significance (Table 3).

**Table 2:** Odds ratio for diabetes complications by miR-146a-5p values [upper quartile (≥ 6.73) vs. lowest quartiles (< 6.73)]

	All complications	CVD	Nephropathy*	Retinopathy
	OR (95% CI)	OR (95% CI)	OR (95% CI)	OR (95% CI)
Model 1	0.41 (0.21–0.79)	0.38 (0.16–0.89)	0.50 (0.24–1.07)	0.41 (0.20–0.84)
Model 2	0.32 (0.14–0.73)	0.32 (0.12–0.84)	0.64 (0.28–1.47)	0.39 (0.16–0.96)
Model 3	0.34 (0.14–0.76)	0.31 (0.11–0.84)	0.72 (0.30–1.71)	0.40 (0.16–0.99)

CVD: cardiovascular diseases

Model 1: adjusted for age, sex, A1c, diabetes duration

Model 2: Model 1 + AER, hypertension, eGFR, smoking

Model 3: Model 2 + NT-proBNP, TNF-α

^*^ models do not include AER

**Table 3:** Odds ratio for all complications by miR-146a-5p values [upper quartile (≥ 6.73) vs. lowest quartiles (< 6.73)] stratified by sex

	Male	Female
	All complications	
	OR (95% CI)	OR (95% CI)
Model 1	0.32 (0.11–0.94)	0.45 (0.19–1.06)
Model 2	0.20 (0.05–0.92)	0.36 (0.13–1.04)
Model 3	0.16 (0.03–0.80)	0.39 (0.13–1.12)

Model 1: adjusted for age, A1c, diabetes duration

Model 2: Model 1 + AER, hypertension, eGFR, smoking

Model 3: Model 2 + NT-proBNP, TNF-α

We also examined the associations between miR-146a-5p levels and individual diabetes complications. As shown in Table 2, results obtained in all cases were predominantly driven by cases with CVD and retinopathy. In Model 1, levels of miR-146a-5p in the upper quartile were associated with 62% and 59% reduced OR for CVD and retinopathy, respectively. The trend of OR changes across models was similar to that observed for all complications and after adjustment for age, sex, A1c, diabetes duration, AER, hypertension, eGFR, and smoking the risk reduction for CVD and retinopathy was 68% and 61%, respectively. After further adjustment for both TNF-α and NT-proBNP the strength of these associations remained statistically significant.

## Discussion

In this case–control study on DM1 patients from the EURODIAB PCS, we showed an independent and inverse association between miR-146a-5p and chronic complications of diabetes.

Values of miR-146a-5p were lower in cases compared to controls. Moreover, in logistic regression analysis, miR-146a-5p levels in the highest quartile distribution of control subjects were associated with a significant risk reduction of diabetic complications, independently of confounders and established risk factors. This suggests that miR-146a-5p may be a candidate marker of vascular protection in DM1. miRNA-based biomarker efficacy may differ between sexes and previous studies reported lower circulating miR-146a-5p levels in women than in men [28, 29] and an influence of sex adjustment on the relationship between circulating miR-146a-5p levels and clinical outcomes [30, 31]. In our study, miR-146a-5p levels did not differ in men and women and adjustment for sex did not modify the results. However, when analyses were performed separately in men and women, the inverse relation between miR-146a-5p levels and diabetes complications was confirmed in men, while it did not reach statistical significance in women. The reason for this gender-specific difference is unknown; however, it may be related to the confounding effect of miR-146a-5p negative modulation by estrogens [32, 33].

Previous studies have reported an age-related decline in circulating miR-146a-5p levels [28, 34]. However, in our study, miR-146a-5p levels did not correlate with age and did not differ across age categories. Moreover, adjustment for age did not modify the results. The reason for this discrepancy is unknown; however, the effect of age on miR-146a-5p levels was observed in very old subjects (> 75 years) [34] and our DM1 patients were relatively young. Moreover, the age-related decline in miR-146 was weaker [34] or even absent [35] in studies performed on patients with type 2 diabetes.

The source of serum miR-146a-5p was not established in our study; however, endothelial cells, platelets, and immune/inflammatory cells are likely candidates [36, 37]. In these cell types, miR-146a-5p expression can be modulated by diabetes. Indeed, exposure of endothelial cells to high glucose induces a persistent miR-146a-5p downregulation [38–40]. Moreover, diabetes induces an inflammatory response that can trigger a compensatory expression of miR-146a-5p. Therefore, the relationship between miR-146a-5p and diabetes is complex and is likely affected by many other variables besides hyperglycemia. This may explain why in our study there was no correlation between miR-146a-5p and A1c, despite the inverse association between miR-146a-5p and diabetes complications.

The biological mechanism underlying the independent and inverse association between serum miR-146a-5p levels and DM1 complications is unknown. However, low serum miR-146a-5p level may mirror a miR-146a-5p downregulation in endothelial cells, monocytes, and other cell types relevant to DM1 complications, where miR-146a-5p deficiency impairs the feedback restraints on diabetes-induced inflammation [40], NOX-4-mediated oxidative stress [41], and apoptosis [42]. Therefore, serum miR-146a-5p level may be a circulating marker of the efficacy of the anti-inflammatory response and thus of the susceptibility to develop complications. Consistent with this, miR-146a-5p is a well-established marker of inflammation and is associated with a variety of immune and non-immune inflammatory conditions [14, 43–45], including CVD, Alzheimer’s disease, and type 2 diabetes [34, 46–49].

On the other hand, low serum miR-146a-5p levels may also be due to reduced miR-146a-5p content in circulating EVs. As uptake of miRNA containing EVs can induce phenotypic changes in the recipient cells, this may hamper the systemic control of miR-146a-5p on inflammation and thus favor the development of DM1 complications [50, 51]. Consistent with this, therapeutic strategies delivering nanoparticles loaded with miR-146a-5p have been successfully tested in various inflammatory conditions [14, 52–54].

The magnitude of the association between miR-146a-5p and all complications was greater after adjustment for A1C, diabetes duration, hypertension, AER, eGFR, TNF-α, NT-pro-BNP, suggesting that miR-146a-5p measurement may have an added value over traditional risk factors in identifying patients at enhanced risk of complications. Diabetes-related risk factors induce a low-grade inflammation and they are thus expected to enhance miR-146a-5p expression (13). This may explain their negative confounding effect in the inverse relationship between miR-146a-5p and all complications. Once induced, miR-146a-5p potently inhibits inflammatory processes that contribute to diabetic complication onset [13]. Therefore, higher circulating miR-146a-5p levels may identify the subgroup of patients able to build a more effective miR-146a-5p-mediated anti-inflammatory response and thus less prone to develop complications. Both miR-146 polymorphisms and epigenetic modifications may affect the efficacy of miR-146 negative feedback loop [55].

At variance with our results, a recent study in patients with type 2 diabetes showed that miR-146a-5p levels in circulating CD31^+^EV were greater in individuals with vascular complications compared with subjects without complications [37]. However, miR-146a-5p CD31^+^EV levels were not adjusted for clinical variables that correlated with miR-146a-5p and differed between the two groups. Moreover, miR-146a-5p was measured on circulating CD31^+^EV rather than on serum, making direct comparison [37] between the studies difficult.

Logistic regression analysis carried out for individual complications showed that higher miR-146a-5p levels were associated with a 60% lower OR of diabetic retinopathy. In line with this finding, miR-146a-5p is downregulated in the diabetic retina [38] and intervention strategies that enhance retinal miR-146 levels either by intravitreal injection or overexpression, ameliorate experimental diabetic retinopathy [56, 57]. Besides lowering inflammation, high miR-146a-5p expression may also affect retinopathy by downregulating the proangiogenic HIF-1α-ROBO4 pathway [58].

We also found an independent and inverse association between miR-146a-5p and CVD. Mechanistically, miR-146a-5p acts as a brake on proinflammatory NF-kB signaling in both endothelial cells and monocytes and this together with miR-146a-5p anti-oxidative properties [41] may explain the link between miR-146a-5p and atheroprotection. Consistent with this, treatment with miR-146a-5p is beneficial in animal models of atherosclerosis [59, 60]. Moreover, a miR-146a-5p polymorphism, resulting in lower mature miR-146a-5p production, confers enhanced risk of coronary artery disease in humans [61].

Besides the potential relevance of miR-146a-5p as a biomarker of CVD in diabetes, serum miR-146a-5p may also have direct vascular protective effects. Indeed, serum EV carrying miR-146a-5p can enter into recipient cells and alter the cell phenotype by releasing miR-146a-5p. Consistent with this, EV-mediated transfer of miR-146a-5p has been demonstrated to modulate inflammation and to improve cardiac function after myocardial infarction [62]. Moreover, in mouse models of atherosclerosis, delivery of miR-146a-5p to the endothelium by injection of either exogenous EV or a free miR-146a-5p mimetic not only ameliorated endothelial inflammation, but also reduced atherosclerotic plaque size [59, 63].

MiR-146a-5p has also been involved in the pathogenesis of diabetic nephropathy [15, 18, 64]. However, we did not observe any significant associations between serum miR-146a-5p levels and this microvascular complication of diabetes. The reason is unknown; however, endothelial cells that are a major source of circulating miR-146a-5p may play a less relevant role in this complication than in CVD and retinopathy.

Our study has several limitations. Although EURODIAB is a prospective study, baseline serum samples were not available; therefore, miR-146a-5p could only be measured in follow-up samples. Given the cross-sectional design of the study, our results only demonstrate the presence of an independent association between miR-146a-5p and chronic diabetes, but they do not prove a clinical relevance of miR-146a-5p as a biomarker of DM1 complications. Moreover, the cross-sectional design of the study restricts our ability to investigate causal relationships and to elucidate mechanisms. However, pilot cross-sectional studies on large and well-characterized existing cohorts are an effective manner to identify miRNAs of potential interest that can be then tested in longitudinal studies. The smaller number of controls compared to cases has reduced the power of the analyses; however, the number of controls and cases with individual complications was similar, though multiple comparisons might have caused significant results due to chance. Lack of matching between cases and controls for age represents a limitation of the study as age can affect circulating miR-146a-5p levels [28, 34]; however, adjustments for age were made at the analysis stage. The study did not include a control group of non-diabetic patients as the EURODIAB study exclusively recruited DM1 patients; however, the major purpose of the study was to assess if miR-146a-5p was associated with diabetes complications rather than compare circulating miR-146a-5p levels in patients with and without diabetes. Results were not adjusted for residual β-cell function; however, the role of C-peptide as marker/therapy for chronic DM1 complications is still controversial. Moreover, C-peptide is usually detectable in the first decade of DM1 [65] and in our study only 10% of patients had a diabetes duration below 10 years. The possibility of sample degradation over time cannot be ruled out. On the other hand, samples were properly stored and miRNAs are stable in biofluids. Although the TRIZOL method for miRNA extraction may be less sensitive compared to specific miRNA extraction kit, there is no an optimal “gold standard” method for isolating miRNAs [66], the TRIZOL method is widely used, performed well in our hands, and is particularly suitable for routine clinical application given its low cost. We acknowledge that there was a considerable variability in Cel-miR-39 Ct values; however, miR-146 data were normalised using both the endogenous control (U6) and the exogenous control (Cel-miR-39). An important strength of our study is the large sample size and the possibility to adjust for the potential confounding effect of other risk factors and complications. Moreover, patients were from a representative sample of European DM1 patients, and results are, thus generalizable.

## Conclusions

In conclusion, in patients with DM1, serum miR-146a-5p levels are independently associated with diabetic complications and in particular with CVD and retinopathy. Prospective studies are warranted to establish if serum miR-146a-5p can be exploited as a clinical biomarker to predict outcomes.

## Supplementary Information

**Additional file 1: Table S1.** Mean Ct values for miR-146a-5p, U6 snRNA and Cel-miR-39 in both controls and cases.

**Additional file 2: Table S2.** Pearson correlation coefficient of clinical variables.

## Data Availability

The datasets used and/or analysed during the current study are available from the corresponding author on reasonable request.

## Abbreviations

PCS: Prospective Complications Study
miR: MicroRNA
DM1: Type 1 diabetes
qPCR: Quantitative PCR
OR: Odds ratio
CI: Confidence interval
A1c: Glycated hemoglobin
AER: Albumin excretion rate
eGFR: Estimated glomerular filtration rate
NT-proBNP: B-type natriuretic peptide
TNF-α: Tumor necrosis factor alpha
CVD: Cardiovascular disease
TGF-β1: Transforming growth factor beta 1
Nf-κB: Nuclear factor kappa-light-chain-enhancer of activated B cells
BMI: Body mass index
IL-6: Interleukin 6
ELISA: Enzyme-linked immunosorbent assay
WHR: Waist to hip ratio
CAD: Coronary artery disease
EV: Extracellular vesicle
HIF-1α: Hypoxia-inducible factor 1 alpha
LDL: Low-density lipoprotein
HDL: High-density lipoprotein
SBP: Systolic blood pressure
DBP: Diastolic blood pressure
sCr: Serum creatinine
Cr Cl: Creatinine clearance
