# Supplementary material for: MicroRNA 146a is associated with diabetic complications in type 1 diabetic patients from the EURODIAB PCS
Source: J Transl Med. 2021 Nov 25;19:475. doi: 10.1186/s12967-021-03142-4 (PMC8614036; doi:10.1186/s12967-021-03142-4)
Supplement: Supplementary file 1 — Additional file 1: Table S1. Mean Ct values for miR-146a-5p, U6 snRNA and Cel-miR-39 in both controls and cases. [file 12967_2021_3142_MOESM1_ESM.docx]

**Table S1.** Mean Ct values for miR-146a-5p, U6 snRNA and Cel-miR-39 in both controls and cases

|  | miR-146a-5p | | U6 snRNA | | Cel-miR-39 |  |
| --- | --- | --- | --- | --- | --- | --- |
| Controls |  | |  | |  |  |
| 1 | 21.55 | | 25.55 | | 17.61 |  |
| 2 | 21.28 | | 23.48 | | 23.41 |  |
| 3 | 23.42 | | 25.88 | | 16.63 |  |
| 4 | 21.06 | | 20.69 | | 20.49 |  |
| 5 | 19.25 | | 25.51 | | 20.46 |  |
| 6 | 23.57 | | 20.80 | | 14.16 |  |
| 7 | 18.95 | | 27.87 | | 13.05 |  |
| 8 | 17.99 | | 26.02 | | 21.93 |  |
| 9 | 17.61 | | 27.21 | | 19.03 |  |
| 10 | 19.71 | | 25.52 | | 16.81 |  |
| 11 | 21.68 | | 26.80 | | 15.46 |  |
| 12 | 20.82 | | 20.22 | | 16.36 |  |
| 13 | 23.11 | | 24.66 | | 16.44 |  |
| 14 | 20.14 | | 24.38 | | 16.84 |  |
| 15 | 21.65 | | 25.24 | | 18.61 |  |
| 16 | 22.99 | | 22.82 | | 15.83 |  |
| 17 | 19.63 | | 25.52 | | 24.05 |  |
| 18 | 19.95 | | 25.87 | | 16.82 |  |
| 19 | 21.75 | | 26.12 | | 18.31 |  |
| 20 | 18.70 | | 26.57 | | 16.46 |  |
| 21 | 23.51 | | 21.69 | | 15.98 |  |
| 22 | 21.73 | | 27.23 | | 15.95 |  |
| 23 | 21.59 | | 24.41 | | 15.80 |  |
| 24 | 22.53 | | 20.66 | | 19.31 |  |
| 25 | 22.24 | | 23.00 | | 19.31 |  |
| 26 | 25.68 | | 25.51 | | 24.61 |  |
| 27 | 22.87 | | 24.55 | | 13.96 |  |
| 28 | 17.26 | | 24.69 | | 13.53 |  |
| 29 | 23.79 | | 25.06 | | 20.06 |  |
| 30 | 22.60 | | 28.05 | | 21.10 |  |
| 31 | 20.63 | | 24.18 | | 16.69 |  |
| 32 | 21.93 | | 26.38 | | 15.27 |  |
| 33 | 24.53 | | 22.03 | | 22.81 |  |
| 34 | 26.80 | | 30.36 | | 19.87 |  |
| 35 | 21.25 | | 21.05 | | 20.26 |  |
| 36 | 21.21 | | 25.19 | | 16.55 |  |
| 37 | 21.40 | | 25.14 | | 23.85 |  |
| 38 | 22.58 | | 20.62 | | 18.43 |  |
| 39 | 22.23 | | 24.21 | | 17.89 |  |
| 40 | 22.14 | | 24.34 | | 21.45 |  |
| 41 | 21.04 | | 23.10 | | 19.05 |  |
| 42 | 20.57 | | 21.91 | | 16.90 |  |
| 43 | 20.49 | | 24.29 | | 26.36 |  |
| 44 | 19.89 | | 25.42 | | 17.96 |  |
| 45 | 20.59 | | 22.87 | | 21.73 |  |
| 46 | 21.45 | | 23.21 | | 24.35 |  |
| 47 | 20.66 | | 28.72 | | 27.80 |  |
| 48 | 20.47 | | 22.35 | | 17.85 |  |
| 49 | 20.13 | | 25.11 | | 17.23 |  |
| 50 | 20.19 | | 21.20 | | 14.06 |  |
| 51 | 20.49 | | 24.29 | | 26.36 |  |
| 52 | 18.28 | | 22.91 | | 16.20 |  |
| 53 | 22.02 | | 21.77 | | 16.07 |  |
| 54 | 21.37 | | 32.62 | | 26.57 |  |
| 55 | 20.63 | | 22.57 | | 11.94 |  |
| 56 | 22.15 | | 23.67 | | 22.17 |  |
| 57 | 22.13 | | 24.20 | | 18.17 |  |
| 58 | 19.02 | | 16.31 | | 22.72 |  |
| 59 | 22.37 | | 21.23 | | 24.37 |  |
| 60 | 23.18 | | 25.76 | | 16.84 |  |
| 61 | 22.08 | | 25.44 | | 16.44 |  |
| 62 | 22.61 | | 26.45 | | 13.86 |  |
| 63 | 23.35 | | 25.16 | | 14.25 |  |
| 64 | 23.61 | | 25.89 | | 18.76 |  |
| 65 | 23.96 | | 31.42 | | 22.16 |  |
| 66 | 25.29 | | 23.49 | | 17.89 |  |
| 67 | 23.82 | | 28.92 | | 18.65 |  |
| 68 | 22.59 | | 21.08 | | 19.16 |  |
| 69 | 18.58 | | 24.26 | | 16.00 |  |
| 70 | 20.19 | | 28.10 | | 16.24 |  |
| 71 | 32.35 | | 31.97 | | 28.67 |  |
| 72 | 24.32 | | 26.53 | | 19.71 |  |
| 73 | 21.04 | | 23.28 | | 18.59 |  |
| 74 | 23.58 | | 32.74 | | 27.19 |  |
| 75 | 17.58 | | 25.43 | | 14.26 |  |
| 76 | 20.52 | | 24.23 | | 17.94 |  |
| 77 | 20.72 | | 19.98 | | 19.00 |  |
| 78 | 22.94 | | 25.53 | | 24.60 |  |
| 79 | 21.25 | | 23.76 | | 17.61 |  |
| 80 | 23.00 | | 27.57 | | 27.67 |  |
| 81 | 22.49 | | 24.71 | | 17.66 |  |
| 82 | 17.92 | | 26.14 | | 17.76 |  |
| 83 | 23.82 | | 29.99 | | 26.58 |  |
| 84 | 18.66 | | 28.62 | | 12.66 |  |
| 85 | 19.06 | | 27.83 | | 16.30 |  |
| 86 | 22.14 | | 20.68 | | 19.11 |  |
| 87 | 18.49 | | 23.58 | | 20.73 |  |
| 88 | 22.30 | | 29.62 | | 24.68 |  |
| 89 | 19.91 | | 23.37 | | 16.61 |  |
| 90 | 22.90 | | 26.93 | | 15.16 |  |
| 91 | 19.31 | | 26.64 | | 13.75 |  |
| 92 | 23.10 | | 22.82 | | 19.97 |  |
| 93 | 19.94 | | 18.34 | | 14.76 |  |
| 94 | 20.71 | | 24.98 | | 22.06 |  |
| 95 | 28.20 | | 23.97 | | 18.01 |  |
| 96 | 17.48 | | 19.37 | | 15.19 |  |
| 97 | 24.01 | | 26.48 | | 16.92 |  |
| 98 | 21.27 | | 21.68 | | 17.81 |  |
| 99 | 21.91 | | 26.37 | | 23.21 |  |
| 100 | 19.51 | | 25.47 | | 15.71 |  |
| 101 | 17.94 | | 24.14 | | 15.98 |  |
| 102 | 19.29 | | 21.06 | | 16.81 |  |
| 103 | 16.03 | | 19.45 | | 21.67 |  |
| 104 | 22.69 | | 24.52 | | 14.65 |  |
| 105 | 20.97 | | 20.07 | | 17.77 |  |
| 106 | 22.18 | | 24.56 | | 19.97 |  |
| 107 | 13.60 | | 18.86 | | 21.91 |  |
| 108 | 19.41 | | 29.38 | | 18.84 |  |
| 109 | 19.59 | | 26.32 | | 17.00 |  |
| 110 | 20.88 | | 31.54 | | 20.85 |  |
| 111 | 18.07 | | 20.92 | | 19.68 |  |
| 112 | 15.60 | | 18.86 | | 21.91 |  |
| 113 | 20.14 | | 24.82 | | 22.39 |  |
| 114 | 21.95 | | 25.46 | | 17.52 |  |
| 115 | 15.84 | | 24.46 | | 13.26 |  |
| 116 | 22.38 | | 30.67 | | 22.74 |  |
| 117 | 20.24 | | 22.52 | | 17.94 |  |
| 118 | 19.50 | | 21.82 | | 16.41 |  |
| 119 | 18.53 | | 24.80 | | 14.01 |  |
| 120 | 14.71 | | 18.24 | | 12.70 |  |
| 121 | 22.61 | | 32.57 | | 28.19 |  |
| 122 | 22.72 | | 24.01 | | 22.61 |  |
| 123 | 20.37 | | 22.25 | | 20.43 |  |
| 124 | 24.27 | | 27.58 | | 28.31 |  |
| 125 | 18.76 | | 23.10 | | 17.70 |  |
| 126 | 22.45 | | 22.76 | | 17.93 |  |
| 127 | 29.96 | | 28.03 | | 21.12 |  |
| 128 | 21.90 | | 24.79 | | 16.76 |  |
| 129 | 18.40 | | 24.07 | | 19.72 |  |
| 130 | 20.11 | | 21.18 | | 21.57 |  |
| 131 | 19.78 | | 24.51 | | 18.63 |  |
| 132 | 16.53 | | 21.80 | | 12.53 |  |
| 133 | 20.60 | | 27.47 | | 19.27 |  |
| 134 | 23.34 | | 24.61 | | 18.78 |  |
| 135 | 24.67 | | 27.50 | | 18.67 |  |
| 136 | 21.78 | | 25.27 | | 22.53 |  |
| 137 | 19.85 | | 24.82 | | 21.60 |  |
| 138 | 21.01 | | 24.21 | | 16.44 |  |
| 139 | 21.96 | | 30.12 | | 29.55 |  |
| 140 | 18.48 | | 26.55 | | 17.93 |  |
| 141 | 23.44 | | 27.52 | | 18.01 |  |
| 142 | 17.44 | | 23.87 | | 17.65 |  |
| 143 | 24.20 | | 26.45 | | 17.74 |  |
| 144 | 20.92 | | 22.59 | | 17.83 |  |
| 145 | 19.64 | | 23.74 | | 15.96 |  |
| 146 | 18.67 | | 17.71 | | 14.96 |  |
| 147 | 21.43 | | 26.22 | | 21.65 |  |
| 148 | 20.16 | | 22.61 | | 21.86 |  |
| 149 | 22.61 | | 29.17 | | 30.98 |  |
| 150 | 17.48 | | 23.51 | | 22.80 |  |
| 151 | 19.89 | | 20.78 | | 17.75 |  |
| 152 | 27.78 | | 31.90 | | 31.90 |  |
| 153 | 15.43 | | 22.78 | | 13.25 |  |
| Cases |  | |  | |  |  |
| 1 | 20.11 | | 23.40 | | 19.41 |  |
| 2 | 21.76 | | 24.58 | | 18.82 |  |
| 3 | 21.91 | | 25.10 | | 11.75 |  |
| 4 | 16.47 | | 22.43 | | 15.05 |  |
| 5 | 16.90 | | 23.24 | | 15.07 |  |
| 6 | 23.24 | | 27.16 | | 22.44 |  |
| 7 | 20.23 | | 26.51 | | 14.82 |  |
| 8 | 22.73 | | 26.58 | | 17.50 |  |
| 9 | 19.86 | | 21.85 | | 17.89 |  |
| 10 | 25.86 | | 24.83 | | 19.67 |  |
| 11 | 22.40 | | 20.68 | | 19.22 |  |
| 12 | 24.88 | | 25.22 | | 14.36 |  |
| 13 | 25.49 | | 24.59 | | 17.21 |  |
| 14 | 21.75 | | 24.89 | | 16.64 |  |
| 15 | 23.47 | | 19.34 | | 18.51 |  |
| 16 | 20.97 | | 25.12 | | 16.39 |  |
| 17 | 25.65 | | 31.16 | | 27.60 |  |
| 18 | 26.34 | | 24.03 | | 19.30 |  |
| 19 | 21.93 | | 21.61 | | 21.52 |  |
| 20 | 21.98 | | 21.39 | | 16.93 |  |
| 21 | 26.92 | | 21.39 | | 23.37 |  |
| 22 | 22.91 | | 30.40 | | 19.20 |  |
| 23 | 25.06 | | 29.85 | | 19.96 |  |
| 24 | 22.53 | | 24.43 | | 17.80 |  |
| 25 | 22.03 | | 20.43 | | 18.10 |  |
| 26 | 22.58 | | 23.74 | | 19.98 |  |
| 27 | 26.43 | | 27.08 | | 19.13 |  |
| 28 | 20.24 | | 23.90 | | 19.45 |  |
| 29 | 23.23 | | 28.36 | | 18.08 |  |
| 30 | 20.45 | | 25.30 | | 16.45 |  |
| 31 | 22.10 | | 21.40 | | 16.28 |  |
| 32 | 22.27 | | 23.90 | | 17.09 |  |
| 33 | 22.43 | | 28.37 | | 19.36 |  |
| 34 | 22.23 | | 21.38 | | 17.31 |  |
| 35 | 24.43 | | 31.13 | | 14.31 |  |
| 36 | 20.79 | | 26.79 | | 17.37 |  |
| 37 | 21.36 | | 26.88 | | 20.34 |  |
| 38 | 21.26 | | 24.52 | | 19.45 |  |
| 39 | 20.97 | | 20.52 | | 17.29 |  |
| 40 | 25.10 | | 28.67 | | 25.86 |  |
| 41 | 22.82 | | 21.46 | | 22.57 |  |
| 42 | 18.08 | | 25.64 | | 18.36 |  |
| 43 | 19.68 | | 27.98 | | 16.11 |  |
| 44 | 22.33 | | 21.45 | | 18.52 |  |
| 45 | 23.72 | | 25.02 | | 17.64 |  |
| 46 | 24.69 | | 25.86 | | 26.32 |  |
| 47 | 21.04 | | 23.74 | | 19.82 |  |
| 48 | 26.57 | | 26.96 | | 27.26 |  |
| 49 | 19.62 | | 23.97 | | 18.51 |  |
| 50 | 22.27 | | 25.02 | | 15.52 |  |
| 51 | 21.74 | | 25.90 | | 16.97 |  |
| 52 | 27.92 | | 32.62 | | 26.36 |  |
| 53 | 19.12 | | 27.21 | | 20.00 |  |
| 54 | 27.97 | | 32.09 | | 31.55 |  |
| 55 | 23.79 | | 23.64 | | 18.35 |  |
| 56 | 21.43 | | 26.84 | | 16.74 |  |
| 57 | 15.50 | | 21.37 | | 16.02 |  |
| 58 | 22.23 | | 26.23 | | 16.00 |  |
| 59 | 23.81 | | 25.81 | | 20.46 |  |
| 60 | 23.15 | | 26.32 | | 19.83 |  |
| 61 | 21.13 | | 20.41 | | 21.63 |  |
| 62 | 21.83 | | 22.88 | | 25.32 |  |
| 63 | 22.13 | | 28.69 | | 17.15 |  |
| 64 | 24.87 | | 28.68 | | 22.86 |  |
| 65 | 24.30 | | 29.85 | | 24.90 |  |
| 66 | 24.03 | | 24.07 | | 20.55 |  |
| 67 | 15.49 | | 25.68 | | 18.92 |  |
| 68 | 23.40 | | 23.56 | | 22.88 |  |
| 69 | 18.34 | | 21.66 | | 13.28 |  |
| 70 | 21.30 | | 21.10 | | 16.92 |  |
| 71 | 21.41 | | 24.90 | | 24.90 |  |
| 72 | 21.58 | | 22.21 | | 19.17 |  |
| 73 | 20.50 | | 22.27 | | 18.09 |  |
| 74 | 24.54 | | 26.69 | | 19.28 |  |
| 75 | 24.04 | | 26.58 | | 22.08 |  |
| 76 | 24.87 | | 25.20 | | 17.59 |  |
| 77 | 21.59 | | 25.68 | | 15.10 |  |
| 78 | 21.57 | | 25.51 | | 19.46 |  |
| 79 | 20.71 | | 22.44 | | 19.42 |  |
| 80 | 24.27 | | 26.27 | | 16.50 |  |
| 81 | 23.25 | | 21.73 | | 18.13 |  |
| 82 | 18.75 | | 22.23 | | 18.35 |  |
| 83 | 23.67 | | 25.46 | | 28.88 |  |
| 84 | 22.09 | | 22.63 | | 18.05 |  |
| 85 | 18.60 | | 28.91 | | 25.72 |  |
| 86 | 18.86 | | 25.75 | | 17.22 |  |
| 87 | 34.77 | | 33.11 | | 30.02 |  |
| 88 | 19.82 | | 21.91 | | 17.95 |  |
| 89 | 21.39 | | 28.44 | | 22.21 |  |
| 90 | 20.35 | | 20.76 | | 17.27 |  |
| 91 | 18.86 | | 24.36 | | 18.40 |  |
| 92 | 25.54 | | 27.62 | | 25.16 |  |
| 93 | 26.60 | | 22.21 | | 18.38 |  |
| 94 | 20.58 | | 25.71 | | 17.31 |  |
| 95 | 23.99 | | 24.34 | | 17.60 |  |
| 96 | 16.73 | | 23.63 | | 14.27 |  |
| 97 | 18.08 | | 29.32 | | 19.76 |  |
| 98 | 21.58 | | 29.38 | | 25.45 |  |
| 99 | 20.76 | | 25.20 | | 18.51 |  |
| 100 | 22.85 | | 20.75 | | 22.66 |  |
| 101 | 31.74 | | 33.82 | | 30.99 |  |
| 102 | 19.67 | | 23.19 | | 20.83 |  |
| 103 | 17.32 | | 23.36 | | 15.63 |  |
| 104 | 19.65 | | 22.30 | | 16.97 |  |
| 105 | 26.66 | | 27.75 | | 22.23 |  |
| 106 | 27.30 | | 22.13 | | 27.81 |  |
| 107 | 21.79 | | 26.81 | | 18.24 |  |
| 108 | 22.48 | | 23.55 | | 16.00 |  |
| 109 | 23.22 | | 23.99 | | 18.53 |  |
| 110 | 21.60 | | 29.47 | | 22.54 |  |
| 111 | 18.85 | | 22.20 | | 16.18 |  |
| 112 | 23.67 | | 28.05 | | 20.37 |  |
| 113 | 21.10 | | 26.53 | | 14.72 |  |
| 114 | 21.97 | | 29.31 | | 13.90 |  |
| 115 | 23.63 | | 26.49 | | 15.05 |  |
| 116 | 23.64 | | 25.43 | | 25.68 |  |
| 117 | 26.18 | | 28.62 | | 20.19 |  |
| 118 | 23.06 | | 23.66 | | 19.07 |  |
| 119 | 21.63 | | 24.35 | | 16.20 |  |
| 120 | 23.19 | | 23.14 | | 16.84 |  |
| 121 | 24.48 | | 25.53 | | 18.25 |  |
| 122 | 30.65 | | 34.50 | | 33.33 |  |
| 123 | 22.50 | | 23.86 | | 24.45 |  |
| 124 | 21.51 | | 25.22 | | 16.67 |  |
| 125 | 21.31 | | 22.36 | | 16.96 |  |
| 126 | 21.68 | | 20.90 | | 18.38 |  |
| 127 | 21.78 | | 24.38 | | 20.08 |  |
| 128 | 21.21 | | 29.26 | | 21.08 |  |
| 129 | 20.90 | | 26.93 | | 15.75 |  |
| 130 | 24.60 | | 26.24 | | 16.22 |  |
| 131 | 23.18 | | 24.09 | | 23.56 |  |
| 132 | 24.39 | | 25.18 | | 20.43 |  |
| 133 | 22.24 | | 26.52 | | 16.20 |  |
| 134 | 19.36 | | 26.71 | | 13.15 |  |
| 135 | 28.00 | | 34.02 | | 34.76 |  |
| 136 | 18.38 | | 23.50 | | 23.39 |  |
| 137 | 22.56 | | 28.43 | | 22.95 |  |
| 138 | 23.52 | | 24.11 | | 22.83 |  |
| 139 | 21.46 | | 21.47 | | 20.86 |  |
| 140 | 22.77 | | 23.72 | | 21.11 |  |
| 141 | 25.96 | | 30.13 | | 28.93 |  |
| 142 | 17.57 | | 23.45 | | 18.95 |  |
| 143 | 18.07 | | 24.03 | | 12.96 |  |
| 144 | 21.98 | | 22.61 | | 21.34 |  |
| 145 | 23.59 | | 25.04 | | 18.21 |  |
| 146 | 23.87 | | 30.01 | | 29.01 |  |
| 147 | 21.92 | | 23.91 | | 16.60 |  |
| 148 | 21.97 | | 23.33 | | 22.08 |  |
| 149 | 22.42 | | 23.09 | | 18.62 |  |
| 150 | 20.14 | | 25.39 | | 10.62 |  |
| 151 | 27.25 | | 23.93 | | 25.48 |  |
| 152 | 23.18 | | 25.41 | | 18.82 |  |
| 153 | 23.52 | | 28.51 | | 16.02 |  |
| 154 | 22.65 | | 21.97 | | 18.20 |  |
| 155 | 23.59 | | 26.50 | | 17.70 |  |
| 156 | 25.00 | | 27.05 | | 23.38 |  |
| 157 | 18.66 | | 21.97 | | 17.75 |  |
| 158 | 20.77 | | 29.35 | | 17.97 |  |
| 159 | 31.35 | | 31.78 | | 32.48 |  |
| 160 | 23.24 | | 23.83 | | 21.22 |  |
| 161 | 21.67 | | 23.31 | | 18.02 |  |
| 162 | 21.89 | | 23.34 | | 16.57 |  |
| 163 | 21.83 | | 23.48 | | 22.05 |  |
| 164 | 20.90 | | 23.78 | | 16.91 |  |
| 165 | 24.81 | | 25.18 | | 22.00 |  |
| 166 | 18.84 | | 24.97 | | 15.40 |  |
| 167 | 20.16 | | 22.16 | | 23.29 |  |
| 168 | 25.10 | | 24.39 | | 28.40 |  |
| 169 | 19.90 | | 24.67 | | 18.61 |  |
| 170 | 24.91 | | 30.62 | | 20.75 |  |
| 171 | 20.38 | | 26.00 | | 15.95 |  |
| 172 | 20.58 | | 24.46 | | 19.81 |  |
| 173 | 19.84 | | 26.98 | | 16.84 |  |
| 174 | 25.13 | | 24.04 | | 18.87 |  |
| 175 | 22.10 | | 26.62 | | 19.03 |  |
| 176 | 21.90 | | 24.17 | | 16.67 |  |
| 177 | 20.09 | | 23.50 | | 17.10 |  |
| 178 | 27.76 | | 27.72 | | 19.85 |  |
| 179 | 22.60 | | 23.85 | | 21.17 |  |
| 180 | 21.82 | | 23.38 | | 19.06 |  |
| 181 | 34.32 | | 26.00 | | 24.14 |  |
| 182 | 23.57 | | 25.56 | | 16.82 |  |
| 183 | 25.17 | | 24.18 | | 20.93 |  |
| 184 | 26.23 | | 26.82 | | 19.06 |  |
| 185 | 23.67 | | 27.42 | | 19.26 |  |
| 186 | 22.96 | | 24.66 | | 16.28 |  |
| 187 | 23.51 | | 27.30 | | 18.71 |  |
| 188 | 18.43 | | 29.24 | | 15.12 |  |
| 189 | 22.03 | | 27.67 | | 17.51 |  |
| 190 | 28.69 | | 30.24 | | 29.48 |  |
| 191 | 22.22 | | 26.74 | | 16.12 |  |
| 192 | 18.28 | | 24.17 | | 17.66 |  |
| 193 | 24.16 | | 27.28 | | 22.67 |  |
| 194 | 21.47 | | 23.69 | | 17.39 |  |
| 195 | 21.17 | | 25.84 | | 18.96 |  |
| 196 | 21.76 | | 21.87 | | 16.31 |  |
| 197 | 20.03 | | 25.15 | | 18.39 |  |
| 198 | 20.78 | | 22.74 | | 15.61 |  |
| 199 | 22.15 | | 27.09 | | 15.36 |  |
| 200 | 21.76 | | 26.60 | | 15.91 |  |
| 201 | 23.09 | | 23.71 | | 16.00 |  |
| 202 | 19.97 | | 24.14 | | 15.62 |  |
| 203 | 23.25 | | 30.47 | | 18.24 |  |
| 204 | 27.66 | | 25.22 | | 18.99 |  |
| 205 | 22.51 | | 27.01 | | 26.91 |  |
| 206 | 21.77 | | 23.11 | | 15.46 |  |
| 207 | 22.94 | | 21.03 | | 20.51 |  |
| 208 | 27.09 | | 30.24 | | 29.63 |  |
| 209 | 19.96 | | 22.80 | | 21.70 |  |
| 210 | 21.18 | | 28.11 | | 18.42 |  |
| 211 | 21.71 | | 24.32 | | 20.55 |  |
| 212 | 26.63 | | 26.99 | | 20.56 |  |
| 213 | 21.87 | | 24.12 | | 15.16 |  |
| 214 | 24.77 | | 29.22 | | 20.76 |  |
| 215 | 19.48 | | 19.56 | | 20.13 |  |
| 216 | 21.83 | | 28.57 | | 18.14 |  |
| 217 | 18.17 | | 16.47 | | 15.60 |  |
| 218 | 24.13 | | 30.62 | | 23.89 |  |
| 219 | 26.38 | | 22.35 | | 23.45 |  |
| 220 | 30.74 | | 26.69 | | 26.83 |  |
| 221 | 16.84 | | 21.95 | | 20.22 |  |
| 222 | 19.63 | | 19.82 | | 15.60 |  |
| 223 | 24.53 | | 28.58 | | 18.13 |  |
| 224 | 22.26 | | 26.69 | | 19.15 |  |
| 225 | 21.99 | | 29.17 | | 14.67 |  |
| 226 | 19.43 | | 21.87 | | 19.04 |  |
| 227 | 24.04 | | 27.62 | | 24.34 |  |
| 228 | 21.56 | | 26.88 | | 15.24 |  |
| 229 | 13.96 | | 24.07 | | 20.07 |  |
| 230 | 24.34 | | 26.42 | | 18.57 |  |
| 231 | 22.58 | | 29.43 | | 24.67 |  |
| 232 | 20.48 | | 23.06 | | 12.45 |  |
| 233 | 23.02 | | 25.14 | | 15.71 |  |
| 234 | 25.77 | | 24.32 | | 25.65 |  |
| 235 | 21.20 | | 25.80 | | 17.83 |  |
| 236 | 22.41 | | 25.78 | | 19.94 |  |
| 237 | 20.07 | | 20.45 | | 16.19 |  |
| 238 | 23.23 | | 32.32 | | 26.92 |  |
| 239 | 20.67 | | 23.72 | | 16.12 |  |
| 240 | 21.31 | | 23.57 | | 17.04 |  |
| 241 | 21.25 | | 19.85 | | 19.77 |  |
| 242 | 21.71 | | 31.10 | | 21.95 |  |
| 243 | 25.97 | | 21.74 | | 20.96 |  |
| 244 | 18.92 | | 22.63 | | 18.62 |  |
| 245 | 23.51 | | 22.61 | | 24.02 |  |
| 246 | 24.06 | | 27.39 | | 21.93 |  |
| 247 | 23.20 | | 28.74 | | 29.32 |  |
| 248 | 23.64 | | 27.69 | | 17.84 |  |
| 249 | 22.17 | | 26.21 | | 16.24 |  |
| 250 | 20.01 | | 24.52 | | 24.53 |  |
| 251 | 24.69 | | 33.85 | | 29.43 |  |
| 252 | 19.79 | | 27.52 | | 16.83 |  |
| 253 | 21.03 | | 24.11 | | 23.38 |  |
| 254 | 21.62 | | 26.23 | | 18.28 |  |
| 255 | 20.06 | | 25.97 | | 16.24 |  |
| 256 | 22.04 | | 28.66 | | 22.28 |  |
| 257 | 20.81 | | 23.35 | | 15.22 |  |
| 258 | 22.61 | | 28.47 | | 16.98 |  |
| 259 | 24.83 | | 25.13 | | 18.93 |  |
| 260 | 21.78 | | 29.39 | | 27.01 |  |
| 261 | 20.16 | | 29.16 | | 17.17 |  |
| 262 | 19.06 | | 29.07 | | 18.27 |  |
| 263 | 20.58 | | 23.71 | | 15.87 |  |
| 264 | 20.19 | | 19.70 | | 16.55 |  |
| 265 | 21.39 | | 25.85 | | 15.75 |  |
| 266 | 19.75 | | 24.48 | | 18.27 |  |
| 267 | 24.44 | | 22.72 | | 19.58 |  |
| 268 | 23.96 | | 22.82 | | 25.87 |  |
| 269 | 19.43 | | 30.22 | | 23.83 |  |
| 270 | 15.79 | | 24.52 | | 13.10 |  |
| 271 | 22.11 | | 24.87 | | 17.22 |  |
| 272 | 19.31 | | 25.91 | | 18.20 |  |
| 273 | 23.37 | | 26.35 | | 22.96 |  |
| 274 | 25.85 | | 24.05 | | 24.10 |  |
| 275 | 20.44 | | 26.22 | | 20.75 |  |
| 276 | 22.68 | | 23.18 | | 23.00 |  |
| 277 | 21.34 | | 26.46 | | 17.90 |  |
| 278 | 21.50 | | 26.61 | | 20.01 |  |
| 279 | 21.40 | | 23.86 | | 18.33 |  |
| 280 | 23.54 | | 25.88 | | 17.98 |  |
| 281 | 21.60 | | 27.49 | | 19.41 |  |
| 282 | 20.03 | | 21.14 | | 16.30 |  |
| 283 | 18.89 | | 24.08 | | 15.98 |  |
| 284 | 21.76 | | 28.20 | | 18.64 |  |
| 285 | 20.03 | | 27.59 | | 19.53 |  |
| 286 | 21.92 | | 29.05 | | 20.26 |  |
| 287 | 29.38 | | 33.90 | | 33.94 |  |
| 288 | 20.10 | | 20.05 | | 14.99 |  |
| 289 | 22.33 | | 22.79 | | 19.79 |  |
| 290 | 20.95 | | 21.44 | | 16.88 |  |
| 291 | 21.56 | | 26.52 | | 19.56 |  |
| 292 | 23.75 | | 21.14 | | 16.93 |  |
| 293 | 25.27 | | 27.09 | | 16.65 |  |
| 294 | 23.14 | | 21.86 | | 16.68 |  |
|  | |  | |  | |  |
|  | |  | |  | |  |
|  | |  | |  | |  |
|  | |  | |  | |  |
|  | |  | |  | |  |
|  | |  | |  | |  |
|  | |  | |  | |  |
|  | |  | |  | |  |
|  | |  | |  | |  |
|  | |  | |  | |  |
|  | |  | |  | |  |
|  | |  | |  | |  |
|  | |  | |  | |  |
