# Supplementary material for: MicroRNA 146a is associated with diabetic complications in type 1 diabetic patients from the EURODIAB PCS
Source: J Transl Med. 2021 Nov 25;19:475. doi: 10.1186/s12967-021-03142-4 (PMC8614036; doi:10.1186/s12967-021-03142-4)
Supplement: Supplementary file 2 — Additional file 2: Table S2. Pearson correlation coefficient of clinical variables. [file 12967_2021_3142_MOESM2_ESM.docx]

**Table S2.** Pearson correlation coefficient of clinical variables

|  | | **miR-146a-5p** | **Age** | **BMI** | **DM Duration** | **SBP** | **DBP** | **A1C** | **T-Chol** | **HDL-Chol** | **LDL-Chol** | **TG** | **eGFR** | **AER** | **TNF-α** | **NT-ProBNP** | **IL-6** |
| --- | --- | --- | --- | --- | --- | --- | --- | --- | --- | --- | --- | --- | --- | --- | --- | --- | --- |
| **miR-146a-5p** | r | 1 | -0.03 | -0.06 | -0.04 | -0.05 | -0.11 | -0.01 | -0.04 | 0.04 | -0.03 | -0.12 | 0.22 | -0.09 | -0.17 | -0.15 | -0.09 |
|  | P |  | 0.543 | 0.197 | 0.436 | 0.259 | 0.019 | 0.792 | 0.413 | 0.454 | 0.588 | 0.010 | 0.000 | 0.055 | 0.000 | 0.002 | 0.07 |
| **Age** | r | -0.03 | 1 | 0.20 | 0.66 | 0.41 | -0.01 | 0.01 | 0.28 | 0.09 | 0.20 | 0.16 | -0.40 | 0.10 | 0.10 | 0.36 | 0.14 |
|  | P | 0.543 |  | 0.000 | 0.000 | 0.000 | 0.789 | 0.755 | 0.000 | 0.045 | 0.001 | 0.000 | 0.000 | 0.044 | 0.029 | 0.000 | 0.002 |
| **BMI** | r | -0.06 | 0.20 | 1 | 0.09 | 0.24 | 0.21 | 0.05 | 0.18 | -0.21 | 0.26 | 0.25 | -0.10 | 0.12 | 0.11 | 0.01 | 0.10 |
|  | P | 0.197 | 0.000 |  | 0.054 | 0.000 | 0.000 | 0.253 | 0.000 | 0.000 | 0.000 | 0.000 | 0.026 | 0.010 | 0.017 | 0.896 | 0.041 |
| **DM Duration** | r | -0.04 | 0.66 | 0,09 | 1 | 0.39 | -0.03 | 0.10 | 0.27 | 0.13 | 0.19 | 0.16 | -0.40 | 0.30 | 0.26 | 0.29 | 0.18 |
|  | P | 0.436 | 0.000 | 0.054 |  | 0.000 | 0.556 | 0.044 | 0.000 | 0.007 | 0.002 | 0.000 | 0.000 | 0.000 | 0.000 | 0.000 | 0.000 |
| **SBP** | r | -0.05 | 0.41 | 0.24 | 0.39 | 1 | 0.57 | 0.09 | 0.30 | -0.02 | 0.32 | 0.24 | -0.41 | 0.42 | 0.29 | 0.34 | 0.12 |
|  | P | 0.259 | 0.000 | 0.000 | 0.000 |  | 0.000 | 0.067 | 0.000 | 0.646 | 0.000 | 0.000 | 0.000 | 0.000 | 0.000 | 0.000 | 0.009 |
| **DBP** | r | **-0.11** | -0.01 | 0.21 | -0.03 | 0.57 | 1 | 0.02 | 0.21 | -0.03 | 0.25 | 0.17 | -0.15 | 0.27 | 0.17 | 0.07 | 0.01 |
|  | P | **0.019** | 0.789 | 0.000 | 0.556 | 0.000 |  | 0.641 | 0.000 | 0.535 | 0.000 | 0.000 | 0.001 | 0.000 | 0.000 | 0.138 | 0.781 |
| **A1C** | r | -0.01 | 0.01 | 0.05 | 0.10 | 0.09 | 0.02 | 1 | 0.16 | -0.08 | 0.14 | 0.26 | -0.09 | 0.37 | 0.26 | 0.06 | 0.20 |
|  | P | 0.792 | 0.755 | 0.253 | 0.044 | 0.067 | 0.641 |  | 0.001 | 0.080 | 0.024 | 0.000 | 0.048 | 0.000 | 0.000 | 0.241 | 0.000 |
| **T-Chol** | r | -0.04 | 0.28 | 0.18 | 0.27 | 0.30 | 0.21 | 0.16 | 1 | 0.16 | 0.94 | 0.44 | -0.26 | 0.28 | 0.22 | 0.22 | -0.01 |
|  | P | 0.413 | 0.000 | 0.000 | 0.000 | 0.000 | 0.000 | 0.001 |  | 0.000 | 0.000 | 0.000 | 0.000 | 0.000 | 0.000 | 0.000 | 0.884 |
| **HDL-Chol** | r | 0.04 | 0.09 | -0.21 | 0.13 | -0.02 | -0.03 | -0.08 | 0.16 | 1 | -0.11 | -0.35 | 0.02 | 0.10 | -0.17 | 0.02 | -0.14 |
|  | P | 0.454 | 0.045 | 0.000 | 0.007 | 0.646 | 0.535 | 0.080 | 0.000 |  | 0.081 | 0.000 | 0.606 | 0.041 | 0.000 | 0.660 | 0.004 |
| **LDL-Chol** | r | -0.33 | 0.20 | 0.26 | 0.19 | 0.32 | 0.25 | 0.14 | 0.94 | -0.11 | 1 | 0.51 | -0.29 | 0.36 | 0.30 | 0.22 | 0.01 |
|  | P | 0.588 | 0.001 | 0.000 | 0.002 | 0.000 | 0.000 | 0.024 | 0.000 | 0.081 |  | 0.000 | 0.000 | 0.000 | 0.000 | 0.000 | 0.095 |
| **TG** | r | **-0.12** | 0.16 | 0.25 | 0.16 | 0.24 | 0.17 | 0.26 | 0.44 | -0.35 | 0.51 | 1 | -0.24 | 0.34 | 0.37 | 0.14 | 0.11 |
|  | P | **0.010** | 0.000 | 0.000 | 0.000 | 0.000 | 0.000 | 0.000 | 0.000 | 0.000 | 0.000 |  | 0.000 | 0.000 | 0.000 | 0.003 | 0.019 |
| **eGFR** | r | **0.22** | -0.40 | -0.10 | -0.40 | -0.41 | -0.15 | -0.09 | -0.26 | 0.02 | -0.29 | -0.024 | 1 | -0.50 | -0.51 | -0.57 | -0.20 |
|  | P | **0.000** | 0.000 | 0.026 | 0.000 | 0.000 | 0.001 | 0.048 | 0.000 | 0.606 | 0.000 | 0.000 |  | 0.000 | 0.000 | 0.000 | 0.000 |
| **AER** | r | -0.09 | 0.10 | 0.12 | 0.30 | 0.42 | 0.27 | 0.37 | 0.28 | 0.10 | 0.36 | 0.34 | -0.50 | 1 | 0.50 | 0.33 | 0.17 |
|  | P | 0.055 | 0.044 | 0.010 | 0.000 | 0.000 | 0.000 | 0.000 | 0.000 | 0.041 | 0.000 | 0.000 | 0.000 |  | 0.000 | 0.000 | 0.000 |
| **TNF-α** | r | **-0.17** | 0.10 | 0.11 | 0.26 | 0.29 | 0.17 | 0.26 | 0.22 | -0.17 | 0.30 | 0.37 | -0.51 | 0.50 | 1 | 0.34 | 0.25 |
|  | P | **0.000** | 0.029 | 0.017 | 0.000 | 0.000 | 0.000 | 0.000 | 0.000 | 0.000 | 0.000 | 0.000 | 0.000 | 0.000 |  | 0.000 | 0.000 |
| **NT-ProBNP** | r | **-0.15** | 0.36 | 0.01 | 0.29 | 0.34 | 0.07 | 0.06 | 0.22 | 0.02 | 0.22 | 0.34 | -0.57 | 0.33 | 0.34 | 1 | 0.22 |
|  | P | **0.002** | 0.000 | 0.896 | 0.000 | 0.000 | 0.138 | 0.241 | 0.000 | 0.660 | 0.000 | 0.000 | 0.000 | 0.000 | 0.000 |  | 0.000 |
| **IL-6** | r | -0.09 | 0.14 | 0.10 | 0.18 | 0.12 | 0.01 | 0.20 | -0.01 | -0.14 | 0.01 | 0.11 | -0.20 | 0.17 | 0.25 | 0.22 | 1 |
|  | P | 0.070 | 0.002 | 0.041 | 0.000 | 0.009 | 0.781 | 0.000 | 0.884 | 0.004 | 0.095 | 0.019 | 0.000 | 0.000 | 0.000 | 0.000 |  |

miR-146a-5p, TG, AER, TNF-α, NT-proBNP and IL-6 data were log-transformed. BMI, body mass index; SBP, systolic blood pressure; DBP, diastolic blood pressure; LDL, low-density lipoprotein; HDL, high-density lipoprotein; TG, triglycerides; eGFR, estimated glomerular filtration rate; AER, albumin excretion rate; NT-proBNP, NT-proB-type Natriuretic Peptide; IL-6, interleukin 6; TNF-α, tumor necrosis factor-alpha.
